# Uncovering key determinants of well-being among older Canadian retirees in New Brunswick: Protocol for a mixed-methods study

**DOI:** 10.1371/journal.pone.0334022

**Published:** 2025-10-10

**Authors:** Yasin M. Yasin, Areej Al-Hamad, Kateryna Metersky, Emily Richard

**Affiliations:** 1 Faculty of Nursing, University of New Brunswick, New Brunswick, Canada; 2 Daphne Cockwell School of Nursing, Faculty of Community Services, Toronto Metropolitan University, Toronto, Canada; 3 Faculty of Nursing, University of New Brunswick, Moncton, New Brunswick, Canada; PLOS: Public Library of Science, UNITED KINGDOM OF GREAT BRITAIN AND NORTHERN IRELAND

## Abstract

**Aims:**

To uncover the key determinants of physical, mental, social, financial, and spiritual well-being among older Canadian retirees living in New Brunswick and explore how individual, community, organizational, and societal factors interact to shape healthier, more fulfilling aging.

**Design:**

Explanatory sequential mixed-methods study guided by the Socio-Ecological Model.

**Methods:**

A cross-sectional survey will be conducted with 600 retirees aged 65 and older using the WISE Scale, a multidimensional measure of well-being. Surveys will be offered online and in person with accessibility supports. Data will be analyzed in SPSS using descriptive statistics and multiple regression. A purposive subsample of 15–25 participants will be invited for semi-structured interviews to enrich understanding of survey findings. Interviews will be thematically analyzed in NVivo, following the four pillars of trustworthiness. Triangulation will integrate quantitative and qualitative findings.

**Discussion:**

The study will generate detailed insights into how multiple layers of influence affect older adults’ well-being in retirement, addressing critical gaps in research, policy, and practice. Findings will inform tailored community programs, effective strategies for healthcare providers, equitable policies, and age-friendly supports that promote holistic well-being for older retirees in New Brunswick, particularly among English-speaking populations. By identifying specific factors that enhance or hinder well-being, this research will support more responsive and inclusive strategies for healthy aging.

## 1. Introduction

Between 1980 and 2021, the global population aged 65 and older nearly tripled, from 260 million to 761 million, and is projected to reach 1.5 billion by 2050, representing about 17% of the global population [1]. Canada mirrors this trend: in 2015, the number of Canadians aged 65 + surpassed those under 15 for the first time [2], and by 2035, nearly one in four Canadians will be 65 or older [3,4]. The shift is especially marked in New Brunswick, where 23% of residents are aged 65 and older making it among the highest provincial proportions and above the national average [4]. Given the province’s aging and largely rural population, understanding older adults’ well-being is increasingly urgent.

These trends present both challenges and opportunities for policy and practice. Older adults contribute meaningfully to families and communities through caregiving, volunteering, and knowledge-sharing [5]. Yet, retirement, often involving withdrawal from structured employment and a shift in social roles, can affect physical, mental, social, financial, and spiritual well-being in diverse ways [6,7].

Retirement, while widely conceptualized as well-earned rest after decades of employment, is far from a uniform experience. Well-being trajectories in retirement vary substantially across individuals and communities. For some, retirement ushers in increased life satisfaction due to greater autonomy, improved work-life balance, and the opportunity to pursue personal interests [8]. For others, the transition can expose vulnerabilities, including financial insecurity, loneliness, and loss of identity, particularly for those who experience involuntary or early retirement [9,10]. Understanding these divergent pathways is vital for fostering equitable, age-friendly environments.

Well-being in aging is multidimensional, extending beyond the absence of illness to include autonomy, life satisfaction, and resilience [11,12]. For retirees, it encompasses physical, mental, social, financial, and spiritual domains [12,13].

Physical well-being in retirement is shaped by lifestyle changes, with studies reporting both increased physical activity [14,15] and heightened sedentary behavior and chronic disease risk due to disrupted routines [16,17]. Individual factors strongly influence these outcomes [18]. Mental well-being may improve through reduced work-related stress for some [8], but others experience anxiety, depression, or cognitive decline [19]. Kitsaki, Katsiroumpa [9] demonstrated that involuntary retirement is especially detrimental to psychological well-being, amplifying risks of loneliness and social withdrawal. Gender differences are evident where women often benefit emotionally from retirement, while men may experience reduced social engagement [20,21].

The social dimension of well-being is shaped by the transition from work-based relationships to community and familial connections. For some retirees, the dissolution of work-centered social networks can lead to decline of social ties [22]. Volunteering and community engagement are protective factors, sustaining a sense of purpose and fostering intergenerational ties [23]. However, barriers such as mobility limitations, lack of transportation, or insufficient community programs can hinder participation. Gender differences further complicate these dynamics; men may experience a steeper decline in social connectedness post-retirement than women, partly due to differences in how work-based networks are replaced by family or community ties [20,21].

Spirituality remains a critical but underexplored domain in retirement research. Evidence suggests that spiritual practices, including religious participation, meditation, or reflection, enhance mental resilience, reduce depression, and contribute to ego integrity [24,25]. In the context of aging, spirituality offers a framework for making sense of life transitions and losses, reinforcing self-worth and social belonging [13]. These practices may serve as coping mechanisms during the transition to retirement, providing a sense of purpose and community support.

Financial well-being is deeply impacted by the shift from employment income to pensions and savings. Between 17% and 50% of retirees face a decline in living standards [26], and gains in financial satisfaction are more common among low-income individuals [27]. Women face disproportionate challenges due to lower lifetime earnings and pension access [28]. While income assistance supports women, men tend to benefit more from investments [28]. Furthermore, inflation further threatens financial security in retirement by eroding the purchasing power of savings. For instance, a $100 purchase in 2013 cost roughly $129 in 2023 [29]. While Old Age Security (OAS) and the Canada Pension Plan (CPP) adjust for inflation, many private pensions do not, leaving retirees -especially those with fixed or limited incomes- financially vulnerable.

### 1.1 Gaps and significance of the current study

This study addresses a critical gap by examining the multidimensional well-being of older Canadian retirees in New Brunswick. In a province with a large rural population and many older adults with histories of precarious or non-unionized work, financial vulnerability is a key concern [30]. Without adequate income support and accessible services, these disparities can cascade into other well-being dimensions, exacerbating health inequities and social exclusion.

While previous studies often focused on individual aspects such as physical or financial health, few have explored how physical, mental, social, financial, and spiritual dimensions interact [19]. This research adopts a holistic approach to capture these interconnections and applies an equity lens to examine how well-being varies by gender, income, and ethnicity. Marginalized groups -such as women [28] and low-income retirees [31], often face compounded challenges. Identifying such disparities will help inform more responsive, person-centered interventions and services. Findings will directly support older retirees by guiding the development of inclusive programs and policies by healthcare providers, community organizations, and decision-makers. The study also contributes to future research aimed at fostering age-friendly, equitable environments for aging populations.

By applying this comprehensive framework, we acknowledge that aging well involves more than health, it includes sustaining social roles, engaging with communities, and nurturing spiritual meaning [24], all shaped by personal resilience and broader structural factors such as pensions and healthcare policies [32,33].

### 1.2 Theoretical farmwork

This study is guided by the **Socio-Ecological Model (SEM)**, a framework that captures the dynamic interplay between personal and environmental factors influencing well-being [33,34]. SEM recognizes that individual outcomes are shaped by interactions across multiple levels: individual, interpersonal, community, organizational, and societal.

At the individual level, factors such as age, gender, health status, financial literacy, and coping mechanisms help explain variations in well-being among retirees. The interpersonal level considers the role of social networks, including family and friends, in providing emotional and practical support. The community level focuses on access to local resources -such as healthcare, recreational programs, and senior centers- that help sustain well-being in later life. The organizational level encompasses policies and programs within workplaces and community groups, such as pre-retirement planning and social initiatives that foster engagement and financial literacy [26]. At the societal level, broader systems -like healthcare, pensions, and social security- influence retirees’ quality of life. Policies ensuring equitable access to care, financial security, and mental health support are particularly important. Using the SEM allows for a holistic and equity-oriented analysis, identifying how structural factors affect diverse groups such as women, low-income individuals, and ethnic minorities. This lens will help uncover gaps in support systems and inform inclusive, targeted policy and program development.

### 1.3 Research objectives

The primary objective of this study is to assess the levels of physical, mental, social, financial, and spiritual well-being among older English-speaking Canadian retirees in New Brunswick. Additionally, the study seeks to identify the key factors influencing these well-being dimensions to better understand the determinants contributing to a fulfilling and healthy retirement. For the purposes of this study, a retiree is defined as an individual aged 65 years or older who is fully retired from their primary paid employment, regardless of whether they receive formal pension income. The following research questions will guide the study:

1. What is the prevalence of physical, mental, social, financial, and spiritual well-being among older Canadian retirees in New Brunswick?
2. Which sociodemographic factors significantly impact the well-being of older Canadian retirees in New Brunswick across these dimensions?
3. How do community-based resources, organizational practices, and broader societal policies influence the physical, mental, social, financial, and spiritual well-being of older Canadian retirees in New Brunswick?

These objectives will generate insights to inform evidence-based policies and community programs, empowering older adults to age well in place.

## 2. Methodology

This study employs an explanatory sequential mixed-methods design, consistent with Creswell and Plano Clark [35] recommendations for studies seeking to both measure prevalence and explain underlying contexts. The design combines a quantitative survey (phase I) with a qualitative interview phase (phase II) to enable a comprehensive exploration of the physical, mental, social, financial, and spiritual well-being of older Canadian retirees in New Brunswick. This design facilitates triangulation and provides deeper insights into the mechanisms shaping older retirees’ well-being within community, organizational, and societal contexts. The study is registered with the Open Science Framework (https://doi.org/10.17605/OSF.IO/PC4GN) to promote transparency, methodological rigor, and reproducibility.

### 2.1 Study setting

The study will be conducted in New Brunswick, Canada; a province with one of the highest proportions of older adults nationally [4]. It has distinct rural demographics, which provide critical context for understanding well-being in less urbanized communities [30]. New Brunswick is also an officially bilingual province (French and English), with approximately 30% of the population identifying French as their first language [36].The current study will focus on the English-speaking population of the province.

### 2.2 Participants

Participants in this study will be older adults who are retired from paid employment and living in New Brunswick. To ensure relevance to the study’s objectives, individuals must be aged 65 years or older and able to provide informed consent and communicate in English. All participants should be fully retired from their main career, reflecting the project’s focus on understanding the determinants of well-being during the retirement transition. Individuals will not be eligible to participate if they are under the age of 65, have not yet retired from their main career, or are unable to participate meaningfully in the survey or interview process due to severe cognitive impairment that would prevent them from providing informed consent or sharing their experiences.

### 2.3 Data collection

Data collection for both the quantitative and qualitative phases is scheduled to begin in September 2025, following disbursement of research funds. Participant recruitment for the quantitative survey is expected to be completed by December 2025, with qualitative interviews concluding by March 2026. Data cleaning and analysis will take place from April to June 2026, and preliminary results are anticipated by July 2026.

#### 2.3.1 Quantitative phase.

Quantitative data will be collected through a structured questionnaire assessing physical, mental, social, financial, and spiritual well-being. Recruitment will occur via partnerships with senior centers and a range of community-serving organizations (e.g., libraries, health centers, multicultural associations, low-income housing, and non-profits) to reach a diverse sample, including racialized and financially insecure older adults. Study information will be shared through posters, word of mouth, email lists, and information sessions in accessible community settings.

To promote accessibility, the survey will be offered in three formats: (1) online via LimeSurvey with QR codes and links; (2) paper copies available at select partner locations; and (3) survey party sessions; group-based, RA-facilitated sessions held in community spaces for participants with barriers related to literacy, vision, technology, or social isolation. RAs will project and read the questions aloud, while participants complete the survey independently using paper or digital devices. Seating will ensure privacy, and RAs will offer neutral assistance upon request. To encourage participation, participants will be entered into a raffle for one of 40 gift cards valued at $50 each.

Well-being will be measured using the WISE Scale (Well-being across Individual, Social, Economic, and Spiritual domains), developed by the principal investigator and adapted for the Canadian context [37]. The 51-item scale was validated using Lawshe [38] method; CVR values ranged from 0.71–1.00 and the overall scale-level CVI was 0.97. The WISE Scale includes five subscales: Physical (10 items), Mental (10), Social (9), Financial (10), and Spiritual well-being (10), rated on a five-point Likert scale from “Strongly Disagree” (1) to “Strongly Agree” (5). A total and subscale score will be calculated. Pilot testing will assess cultural acceptability, and further psychometric analyses (e.g., factor analysis) will validate its use in this context. The questionnaire also captures key sociodemographic variables (e.g., age, gender, marital status, income, education, housing, years since retirement, chronic illness) and includes seven items assessing perceptions of available services for retirees in New Brunswick.

Sample size was calculated using G*Power 3.1.9.2 for multiple regression with 30 predictors, medium effect size (F² = 0.15), α = 0.05, and power = 0.95, yielding a minimum of 260. To enable subgroup analyses (e.g., by gender, income, or community type), validation of study questionnaire, and account for missing data, the target sample size was increased to 600 participants.

#### 2.3.2 Qualitative phase.

Following the quantitative phase, a purposive subsample of approximately 15–25 survey participants will be invited to participate in one-time semi-structured interviews. Sampling will aim for maximum variation across gender, living arrangement, income level, and community type (urban/rural). Final sample size will be guided by data saturation, when no new themes emerge from subsequent interviews.

Interviews will be conducted by a trained research assistant under the supervision of the Principal Investigator (PI). The PI will provide training and observe the first interview. While prior relationships are unlikely, some participants may recognize the interviewer from the survey phase, particularly if they attended a survey party session. Any such interactions would have been brief and limited to survey support. Participants will be informed of the research team’s names, roles, and affiliations in the consent form but will not receive further details about team members’ characteristics.

Interviews will follow a semi-structured guide informed by the SEM framework and preliminary survey findings. Topics will include retirement experiences, well-being across the five WISE domains, and perceived support and barriers at the community and policy level. The guide will be pilot-tested with 1–2 participants and refined if needed. Interviews will be held in private rooms at senior centers or via secure video or phone calls, based on participant preference. Sessions will last approximately 45–60 minutes, be audio-recorded with consent, and transcribed verbatim. Field notes will be taken during or immediately after. Transcripts will not be returned for participant review. Interviewees will receive a $50 gift card honorarium and may use a pseudonym during the interview and on consent forms.

### 2.4 Data management and analysis

All quantitative data will be managed and analyzed using SPSS (version 29). Raw survey responses will be checked for completeness, cleaned, and securely stored in password-protected files accessible only to the research team. Descriptive statistics (including means, standard deviations, frequencies, and percentages) will be used to summarize participant characteristics and describe the levels of physical, mental, social, financial, and spiritual well-being among older retirees. Inferential statistical tests, such as t-tests and one-way ANOVA, will be used to examine group differences based on key sociodemographic variables (e.g., gender, rural vs. urban location, income level). Multiple linear regression analyses will be conducted to identify significant predictors of each well-being domain, controlling for relevant covariates. Assumptions for each test will be checked to ensure validity of results.

Qualitative data from the semi-structured interviews will be transcribed verbatim and analyzed using NVivo software to support systematic coding, organization, and theme development [39]. Braun and Clarke [40] thematic analysis approach will be used, with transcripts read independently by two researchers to identify initial meaning units and develop coding categories. Themes will be identified as derived from the data, not in advance. The study will adhere to the four pillars of trustworthiness in qualitative research: credibility, confirmability, dependability, and transferability [41]. Credibility will be enhanced through peer debriefing, member checking with a subset of five participants, and triangulation with quantitative findings. An audit trail will be maintained to support confirmability and dependability, and detailed contextual descriptions will be provided to support transferability to similar settings.

Finally, a mixed-methods triangulation approach will be employed to integrate quantitative and qualitative findings. The results will be compared and contrasted to identify areas of convergence, complementarity, or divergence, providing a deeper and more nuanced understanding of the key determinants of well-being among older Canadian retirees. This explanatory approach will ensure that the quantitative trends are meaningfully contextualized with participants’ lived experiences and perspectives, strengthening the study’s overall validity and practical implications.

### 2.5 Ethical considerations

This study has received ethics approval from University of New Brunswick Ethics Review Board (REB# 2025−135) and Toronto Metropolitan University Research Ethics Board (REB 2025−362). All participants will provide informed consent in line with institutional and Tri-Council guidelines. Data will be securely stored in password-protected files and destroyed in accordance with institutional policies. Anonymity and confidentiality will be upheld in all publications and presentations.

For the quantitative survey phase, completion of the online survey or submission of a paper survey will constitute informed implied consent. To promote inclusivity, “survey parties” will be hosted at partner centers, where survey questions will be read aloud and projected on screens to support participants who may have physical dexterity issues, literacy or visual barriers. For the qualitative interview phase, written informed consent will be obtained from all participants prior to interviews. Participants may choose to sign their consent form using a pseudonym if they wish to further protect their identity. Audio-recording consent will be explicitly documented on the form.

### 2.6 Dissemination plan

Findings from this study will be shared widely with academic, policy, and community audiences to maximize impact and relevance. Scholarly dissemination will include submission of manuscripts to peer-reviewed journals focused on gerontology, aging, and public health, as well as presentations at national and international conferences. Knowledge mobilization activities will also be tailored for non-academic audiences; these will include policy briefs for decision-makers, plain-language summaries for community organizations, and interactive community forums with partnering senior centers. Participants will be offered summaries of the main findings in accessible formats. This multi-pronged dissemination strategy will ensure that results inform future research, guide policy development, and support community programming aimed at improving the well-being of older Canadian retirees.

## 3. Discussion

Older adults represent one of the fastest-growing demographic groups in Canada [1,4], and understanding the factors that influence their well-being during the transition to and through retirement is increasingly vital. Previous research shows that retirement can affect multiple aspects of well-being — including physical, mental, social, financial, and spiritual dimensions — in complex and sometimes contradictory ways [13,14,42]. However, much of the existing literature focuses on single aspects of well-being or does not fully account for how community, organizational, and policy contexts interact with individual circumstances. This study seeks to address these gaps by applying a comprehensive, mixed-methods approach to uncover the key determinants of well-being among older English-speaking Canadian retirees living in New Brunswick, a province notable for its aging population and predominantly rural communities [30].

By combining quantitative and qualitative methods, this study will generate a nuanced understanding of retirees’ well-being. The quantitative phase will provide a snapshot of prevalence and predictors, while the qualitative phase will illuminate how retirees navigate the challenges and opportunities associated with aging in place. Using the SEM as a guiding framework allows us to examine how individual factors intersect with interpersonal, community, organizational, and societal influences [32,33]. This approach aligns with recommendations from national aging strategies that emphasize the need for age-friendly environments and policies that recognize the diversity of older adults’ experiences [3,43].

Findings from this study will inform the development of targeted programs, services, and policies to promote healthier, more fulfilling aging post-career. For example, insights about disparities in financial security or the importance of spiritual well-being as a protective factor for mental health can support community organizations and senior centers in tailoring programs that address the unique needs of older adults. Moreover, examining how social and community supports buffer the negative impact of retirement aligns with evidence that strong social ties and volunteer activities can sustain well-being in later life.

The study’s explicit focus on equity, diversity, and inclusion will help uncover disparities among different groups of retirees, including those related to gender, income, or rural versus urban residence. Recognizing these differences is critical to ensuring that interventions are accessible, relevant, and equitable. The participatory components of this project, such as working closely with local senior centers and hosting community forums to share and reflect on findings, are intended to enhance the practical impact of the research and support knowledge mobilization. These strategies will not only facilitate meaningful exchange of knowledge but also empower older adults and community stakeholders to co-create solutions that align with local priorities.

Ultimately, this research has the potential to advance knowledge, inform health and social policy, and enhance practice aimed at supporting aging populations. By identifying key determinants of well-being and highlighting opportunities for targeted intervention, the study contributes to the development of age-friendly, equity-focused care environments for older Canadians. The findings will help healthcare providers, policymakers, and community organizations design and implement holistic, person-centered strategies that promote healthier, more fulfilling aging experiences for retirees in New Brunswick. Moreover, this work may serve as a model for similar initiatives in other provinces and global contexts grappling with the complex challenges and opportunities of population aging.
